# Exploring the dual role of endoplasmic reticulum stress in urological cancers: Implications for tumor progression and cell death interactions

**DOI:** 10.1002/ccs3.12054

**Published:** 2024-11-03

**Authors:** Najma Farahani, Mina Alimohammadi, Mehdi Raei, Noushin Nabavi, Amir Reza Aref, Kiavash Hushmandi, Salman Daneshi, Alireza Razzaghi, Afshin Taheriazam, Mehrdad Hashemi

**Affiliations:** ^1^ Farhikhtegan Medical Convergence Sciences Research Center Farhikhtegan Hospital Tehran Medical Sciences Islamic Azad University Tehran Iran; ^2^ Department of Immunology School of Medicine Shahid Beheshti University of Medical Sciences Tehran Iran; ^3^ Health Research Center Life Style Institute Baqiyatallah University of Medical Sciences Tehran Iran; ^4^ Independent Researcher Victoria British Columbia Canada; ^5^ Department of Surgery Massachusetts General Hospital Harvard Medical School Boston Massachusetts USA; ^6^ Nephrology and Urology Research Center Clinical Sciences Institute Baqiyatallah University of Medical Sciences Tehran Iran; ^7^ Department of Public Health School of Health Jiroft University of Medical Sciences Jiroft Iran; ^8^ Social Determinants of Health Research Center Research Institute for Prevention of Non‐Communicable Diseases Qazvin University of Medical Sciences Qazvin Iran; ^9^ Department of Orthopedics Faculty of Medicine Tehran Medical Sciences Islamic Azad University Tehran Iran; ^10^ Department of Genetics Faculty of Advanced Science and Technology Tehran Medical Sciences Islamic Azad University Tehran Iran

**Keywords:** bladder cancer, cell death dynamics, endoplasmic reticulum stress, prostate cancer, renal cancer

## Abstract

The endoplasmic reticulum (ER) is crucial for maintaining calcium balance, lipid biosynthesis, and protein folding. Disruptions in ER homeostasis, often due to the accumulation of misfolded or unfolded proteins, lead to ER stress, which plays a significant role in various diseases, especially cancer. Urological cancers, which account for high male mortality worldwide, pose a persistent challenge due to their incurability and tendency to develop drug resistance. Among the numerous dysregulated biological mechanisms, ER stress is a key factor in the progression and treatment response of these cancers. This review highlights the dual role of aberrant ER stress activation in urologic cancers, affecting both tumor growth and therapeutic outcomes. While ER stress can support tumor growth through pro‐survival autophagy, it primarily inhibits cancer progression via apoptosis and pro‐death autophagy. Interestingly, ER stress can paradoxically aid cancer progression through mechanisms such as exosome‐mediated immune evasion. Additionally, the review examines how pharmacological interventions, particularly with phytochemicals, can stimulate ER stress‐mediated tumor suppression. Key regulators, including PERK, IRE1α, and ATF6, are discussed for their roles in upregulating CHOP levels and triggering apoptosis. In conclusion, a deeper understanding of ER stress in urological cancers not only clarifies the complex interactions between cellular stress and cancer progression but also provides new opportunities for innovative therapeutic strategies.

## Graphical abstract

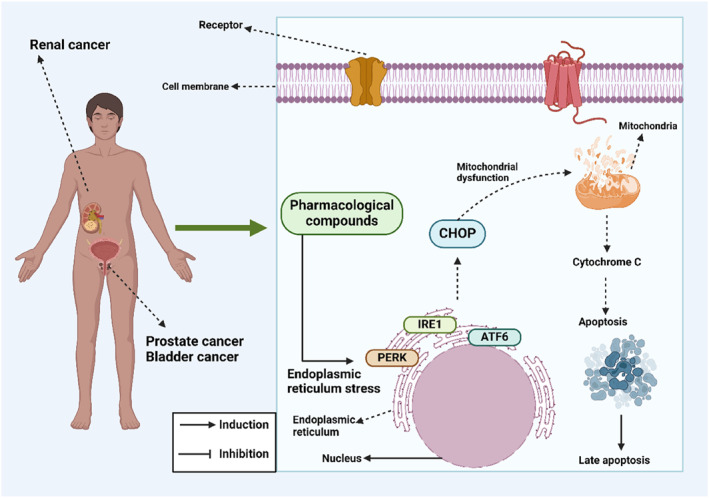

## 1 INTRODUCTION

Cancer is considered one of the most malignant conditions second only to cardiovascular diseases. The development of cancer is a highly complex process with no single factor identified as the sole initiator, highlighting its multifactorial origin. Its etiology remains uncertain, making this multifactorial disease a persistent threat to humans and a main cause of death among patients. Cancer cells differ from normal cells due to their abnormally high proliferation rate, uncontrolled metastasis, and ability to develop drug resistance along with radioresistance and immune evasion capacities. Genomic studies have revealed the involvement of numerous deletions, mutations, and insertions occurring during tumorigenesis. The gradual accumulation of these mutations contributes to the malignancy and aggressiveness of tumors in advanced stages. Additionally, epigenetic factors, particularly noncoding RNAs, play a significant role in carcinogenesis. In recent years, alongside efforts to understand the genomic profile's role in tumor initiation and progression, attention has also shifted toward understanding the role of specific intracellular organelles in cancer progression. A key focus has been the central role of mitochondria in apoptosis and their function during cancer progression.^1^, ^2^, ^3^, ^4^ Research has also increasingly focused on other organelles, particularly the endoplasmic reticulum (ER), which plays a significant role in modulating carcinogenesis. Protein synthesis is essential for life, and when some proteins remain unfolded, harmful aggregates can form.^5^ Although the protein folding process takes place in the ER, it is influenced by mRNA translation and an imbalance in protein folding efficiency with the lipid membrane disrupting the coordination between these two mechanisms.^6^, ^7^ Misfolded or unfolded proteins in the ER cause ER stress.^7^ The cellular stress triggers the activation of ER chaperones, and subsequent studies uncovered what is now known as the unfolded protein response (UPR). The development of the UPR disrupts the ER's protein loading and folding capacity, primarily due to disturbances in the inositol‐requiring enzyme 1 (IRE1), which functions as an ER sensor.^8^ Therefore, understanding the function of ER stress in the disease pathogenesis, especially cancer is of importance.

Since then, two additional ER stress sensors have been identified: activating transcription factor 6 (ATF6) and protein kinase RNA‐like ER kinase (PERK). Both sensors play key roles in regulating ER functions and protein synthesis.

The ER is responsible for synthesizing, folding, and secreting proteins.^9^ The ER is also involved in processes such as Ca2+ buffering and the biosynthesis of lipids and phospholipids. Disruptions in protein folding within the ER can arise from conditions including starvation or viral infections. These triggers activate cellular processes that disrupt protein synthesis, preventing cells from effectively managing stress. This is a consequence of the UPR, which also promotes protein degradation by stimulating autophagy, with the increase in unfolded protein clearance referred to as ER‐associated degradation (ERAD). UPR sensors are in the ER membranes and interact with the chaperone GRP78/BiP in the ER lumen. When GRP78/BiP binds to unfolded proteins in the ER, the cytosolic components of the sensors are autoactivated through transphosphorylation, triggering signaling processes that help maintain homeostasis. Therefore, the three UPR sensors play a crucial role in preserving ER homeostasis.^10^

In recent years, the role of ER stress in regulating cancer progression has been discovered.^11^, ^12^, ^13^, ^14^, ^15^ This review examines the role of ER stress in modulating the progression of urological cancers. It begins by outlining fundamental information about ER stress and its function in cancer. Next, it provides an overview of the three major urological cancers and discusses the involvement of ER stress in drug resistance. Finally, it introduces pharmacological agents that modulate ER stress and enhance cancer treatment outcomes.

## 2 ER STRESS IN CANCER: MOLECULAR BASIS AND ROLE IN CANCER

### 2.1 PERK pathway

PERK, a type I transmembrane protein located in the ER, can suppress mRNA translation to trigger ER stress, with its luminal domain sharing similarities with IRE1.^16^, ^17^, ^18^, ^19^ Under physiological conditions, PERK interacts with the BiP protein, and upon activation, it reduces the influx of newly synthesized proteins into the stressed ER compartment by downregulating eIF2 through phosphorylation at serine 51.^20^ This leads to the downregulation of eIF2B, a guanine nucleotide exchange factor complex responsible for recycling eIF2 into its active GTP‐bound form.^21^ This reduces the overload of misfolded proteins, helping to alleviate ER stress.^22^ Additionally, eIF2 phosphorylation triggers the translation of UPR‐dependent genes, including ATF4, which contains several upstream open reading frames.^23^, ^24^ ATF4 can enhance the expression of ER stress‐related targets, including CHOP, growth arrest, GADD34, and ATF3.^25^, ^26^

### 2.2 IRE1α pathway

IRE1α is the most evolutionarily conserved ER stress sensor.^27^ It is a transmembrane protein consisting of a luminal sensor domain, a cytosolic serine/threonine kinase, and an RNase domain.^28^ Under normal physiological conditions, the luminal domain of IRE1α is bound to the BiP chaperone, preventing its dimerization. However, during moderate ER stress, misfolded or unfolded proteins compete with BiP for binding to IRE1α. This leads to homodimerization and autophosphorylation of IRE1α, activating its endoribonuclease function.^29^ Upregulated IRE1α cleaves a 26‐base intron from XBP1u, resulting in a translational frame‐shift mRNA known as XBP1s.^30^ XBP1s then translocates to the nucleus, where it regulates UPR‐related factors, enhancing protein folding, secretion, and ERAD to manage the accumulation of misfolded proteins in the ER.^31^ The downregulation of CHOP and XBP1 plays a role in cell survival. Under severe ER stress, unfolded proteins can bind to IRE1α, triggering its oligomerization.^32^ This induces cleavage at XBP1‐like sites in ER‐associated mRNA, ribosomal RNA, and miRNAs,^33^ a process known as IRE1‐dependent decay (RIDD), which helps restore ER homeostasis or leads to cell death.^34^ The cytoplasmic domain of IRE1α can bind to tumor necrosis factor receptor‐associated factor 2 (TRAF2), activating ASK1 and JNK. This triggers ER stress‐mediated apoptosis by increasing BIM levels and decreasing Bcl‐2 expression.^35^

### 2.3 ATF6 pathway

ATF6, another ER‐resident type II transmembrane protein, has the following two homologs: ATF6α and ATF6β.^36^ ATF6 serves as a precursor to the cytoplasmic N‐terminal bZIP transcription factor.^37^ After ATF6 dissociates from BiP, ATF6α is transported to the Golgi apparatus via COPII‐coated vesicles, where it is cleaved by site‐1 and site‐2 proteases, activating its function as a transcription factor.^38^ ATF6α regulates genes involved in the folding and glycosylation of newly synthesized proteins, playing a key role in maintaining the viability of stressed cells.^39^ ATF6α shares common targets with XBP1, highlighting the overlap between the ATF6α and IRE1α pathways. While the roles of IRE1α and PERK in response to ER stress are well understood, silencing ATF6 reduces cell survival under ER stress, indicating that ATF6α plays a supportive role in promoting cell viability.^39^

### 2.4 Chaperones

**FIGURE 1:**
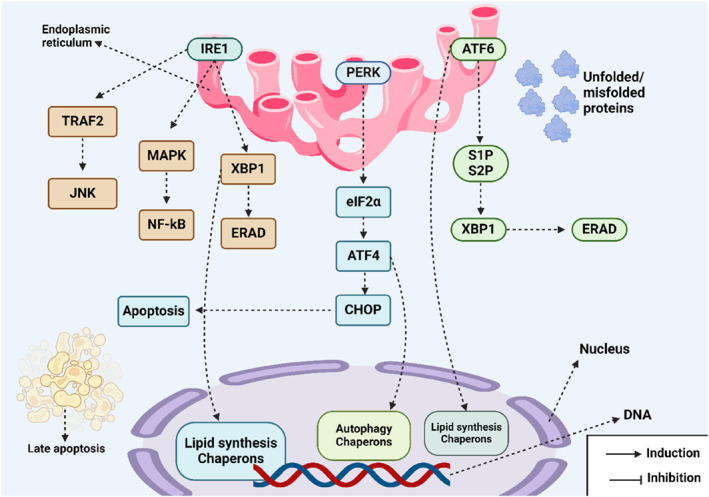
A schematic representation of ER stress in a cell shows the activation of the UPR system, which consists of the following three sensors: ATF6, IRE1, and PERK. IRE1 activates the TRAF2/JNK axis and MAPK/NF‐kB pathway while also promoting the splicing of XBP1 mRNA to induce ERAD. PERK increases eIF2α levels to inhibit protein synthesis and upregulates ATF4 leading to CHOP‐induced apoptosis. ATF6 enhances the activity of S1P and S2P boosting ERAD levels (Biorender.com).^50^, ^51^ ER, endoplasmic reticulum.

GRP78/BiP is the most well‐known and widely recognized chaperone that detects polypeptides with exposed hydrophobic residues.^40^ GRP78/BiP is activated in response to the presence of misfolded or unfolded proteins in the ER.^41^, ^42^ GRP78/BiP recognizes these proteins and assists in refolding them into their native conformations. It also binds to nascent hydrophobic proteins protecting them from aggregation.^43^ If misfolded proteins resist the folding process they are targeted by the ERAD machinery and directed for degradation.^44^ GRP78/BiP plays a crucial role in guiding misfolded proteins for retro‐translocation into the cytosol, where they undergo degradation via the ERAD pathway.^45^, ^46^, ^47^ Additionally, GRP78/BiP maintains the permeability barrier between the cytosol and ER lumen by interacting with the heteromeric Sec61 translocon complex.^48^ Other chaperones similar to GRP78/BiP such as GRP94 and GRP170 also facilitate protein folding, assembly, and transport. They assist in the translocation of misfolded proteins into the cytosol for degradation via ERAD, though their substrate specificities are narrower compared to GRP78/BiP.^49^ Figure 1 provides a schematic representation of ER stress.

### 2.5 ER stress in cancer: Beyond urological cancers

In cancer, ER stress helps tumors adapt to nutrient and oxygen shortages, but it can also initiate apoptosis.^52^ This dual role includes both the UPR and interactions with macrophages, which promote tumor aggressiveness by releasing cytokines and growth factors.^53^ Mahadevan et al.^54^ highlight a significant interaction between macrophages and tumor cells. ER stress pathways increase cyclooxygenase‐2 levels through NF‐κB, promoting cell survival and inflammation while maintaining IL‐8 production.^55^, ^56^ Caspase‐12, located on the ER membrane, specifically initiates apoptosis triggered by ER stress. VEGF‐induced upregulation of Grp78 enhances angiogenesis and growth in endothelial cells, while its inhibition can suppress these processes through MAPK signaling.^57^, ^58^ Additionally, P38MAPK helps maintain cells in a drug‐resistant quiescent state, while PERK‐eIF2α inhibits cell growth, thereby slowing tumor development.^59^, ^60^, ^61^

The UPR pathway has been linked to cancer,^62^ with specific mutations in IRE1α identified across various cancer types.^63^, ^64^ Under hypoxic conditions, the IRE1α pathway's output, XBP1s, promotes tumor survival and growth. It helps cancer cells endure low oxygen levels by forming a transcriptional complex with Hypoxia‐inducible factor (HIF)‐1, a key regulator of genes in hypoxic environments.^65^ This factor is also involved in the initiation and progression of human breast cancer including triple‐negative breast cancer.^65^, ^66^ In a parallel pathway, PERK promotes cell growth and survival through ATF4, a transcription factor that activates the expression of genes essential for cell survival.^67^, ^68^ ATF4 overexpression in solid tumors is crucial for their survival in various mouse and human cancers, and targeting ATF4 for elimination induces apoptosis in cancer cells.^68^ PERK also promotes tumor growth by upregulating vascular endothelial growth factor (VEGF) which drives angiogenesis in tumors. Tumors from PERK‐deficient mouse embryonic fibroblasts exhibit significantly reduced size compared to their wildtype counterparts due to their impaired ability to promote angiogenesis.^67^

## 3 ER STRESS IN PCA

PCa originates from the epithelial compartment of the prostate. The development of the prostate depends on AR signaling which is also essential for maintaining the tissue's physiological function.^69^ The early oncogenic events leading to PCa are not yet fully understood due to its heterogeneous nature and the involvement of numerous oncogenic mechanisms. While hereditary factors contribute to PCa development age remains the primary risk factor. As men age, the following two key changes are observed: a decline in testosterone levels and an increase in proinflammatory cytokines like interleukin‐6 (IL‐6). Systemic inflammation, however, can lower inflammatory cytokine levels in hypogonadal men.^70^ IL‐6 induces changes in the AR cistrome and transcriptome and can activate AR at suboptimal levels. A study involving 218 PCa patients revealed that AR activation occurs in 56% of primary PCa cases increasing to 100% in metastatic PCa.^71^ The emphasis on AR stems from its critical role in the growth, normal development, and function of the prostate. To maintain the physiological function of prostate tissue, the intracellular enzyme 5α‐reductase (5αR) converts circulating testosterone into dihydrotestosterone (DHT) through intracrine mechanisms.^72^ DHT has a strong affinity for binding to AR and is 2 to 10 times more potent than testosterone in activating AR's transcriptional activity.^73^, ^74^ Loss of 5αR can lead to the development of male pseudohermaphroditism while elevated levels of DHT are associated with benign prostatic hyperplasia (BPH) and PCa.^75^, ^76^, ^77^, ^78^ In BPH patients, reducing DHT synthesis by inhibiting 5αR can decrease prostate volume, alleviate symptoms, improve urinary flow rates, and lower the risk of urinary retention or the need for BPH‐related surgery.^79^, ^80^ Ex vivo experiments have demonstrated that DHT is more effective than testosterone in promoting the growth of PCa.^81^ Reducing DHT levels through 5αR downregulation can lower the risk of developing PCa. The findings of the PCa Prevention Trial (PCPT) confirmed the role of 5αR inhibitors as chemopreventive agents in PCa treatment.^82^, ^83^, ^84^

Given that PCa cells depend on androgens for their growth, androgen‐deprivation therapy (ADT) was developed as a treatment strategy.^85^ Advanced PCa patients initially respond to ADT but recurrence occurs due to the presence of castration‐resistant cells. These castration‐resistant PCa (CRPC) cells retain the ability to express androgen‐responsive genes such as prostate‐specific antigen (PSA) and typically continue to express AR.^86^, ^87^ The treatment of localized PCa typically involves the use of external‐beam radiation therapy and radical prostatectomy.^88^, ^89^ These treatment strategies have shown great promise and have successfully improved the survival rate in patients with localized PCa. The prognosis for PCa patients is generally favorable, with a 5‐year survival rate exceeding 90.^90^ However, a challenge remains with recurrence occurring 12–33 months after therapy.^91^ Once CRPC develops, metastasis typically occurs, primarily affecting the axial skeleton. In patients with bone metastasis, therapeutic strategies result in a survival rate of less than 25%.^92^, ^93^, ^94^ Bone metastatic PCa can lead to several complications in advanced cases, such as pathological fractures and spinal cord compression, collectively known as skeletal‐related events. These complications contribute to a poor prognosis in patients.^95^ In such cases, taxane‐based chemotherapy and corticosteroids are used; however, there is a high risk of relapse particularly within the first 5 years.^96^

In recent years, there has been a growing understanding of the molecular mechanisms involved in the progression of PCa. Stress has been identified as a significant contributing factor in this progression, often arising from various cellular influences.^97^, ^98^ Decreased EZH2 expression activates the dsRNA/STING/interferon stress pathway, improving the effectiveness of PD‐1 checkpoint therapy in PCa.^99^ Polymorphisms in oxidative stress‐related mechanisms, such as MAPK14, have been linked to an increased risk of developing PCa.^100^ Additionally, fluid shear stress activates the Piezo1 channel which triggers the Src/YAP axis and promotes PCa invasion.^101^

### 3.1 Progression and drug resistance

ER stress in PCa is tightly regulated with several molecular mechanisms identified for its modulation. Abnormal lipid accumulation has been extensively documented in various cancers including renal, breast, and PCa, among others.^102^, ^103^, ^104^ Lipid accumulation has been linked to the development of CRPC and the emergence of drug resistance.^105^, ^106^ Additionally, lipid metabolism has been associated with ER stress in PCa. Low ACSS3 expression is observed in PCa, indicating a poor prognosis. Methylation of the ACSS3 promoter reduces its expression. Upregulation of ACSS3 can decrease lipid droplet accumulation, trigger ER stress, and reduce androgen synthesis thereby promoting apoptosis, delaying the progression of CRPC, and increasing sensitivity to enzalutamide.^107^ However, the underlying mechanisms linking lipid droplets to the ER stress requires additional investigation. Noteworthy, the noncanonical mechanism of ER stress can accelerate the progression of PCa. The upregulation of S1P and S2P increases the progression of PCa and this is accelerated by the downregulation of GCC185. The fragmentation of Golgi can provide the translocation of S1P and S2P from Golgi to ER to induce cleavage of ATF6. Then, stimulation of UPR occurs to enhance the proliferation of cells. The dissociation of S1P and S2P from Golgi and the upregulation of ATF6 promotes AR signaling. The reduction in ATF6 expression can impair the progression of PCa and reduces the levels of pro‐metastatic factors.^108^ Thus, the role of ER stress is determined by the pathways that regulate it, and in certain cases, it can lead to tumorigenesis. In this context, the downregulation of ATF6 reduces ER stress and Golgi fragmentation, and when combined with autophagy suppression, it contributes to a decrease in PCa tumorigenesis.^109^ However, the role of noncanonical ER stress, or atypical ER stress, does not always promote PCa progression. The use of NSC606985, a camptothecin analog, induces ER stress by increasing the mRNA levels of CHOP, GRP78, and XBP1s. Notably, unlike other compounds, NSC606985 reduces GRP78 protein levels, triggering atypical ER stress‐mediated cell death in PCa.^110^

The EPHB4 receptor tyrosine kinase regulates various biological mechanisms and molecular processes including growth, survival, angiogenesis, vasculogenesis, and neural development.^111^ The interaction between EPHB4 and Ephrin B2 can trigger bidirectional signaling.^111^ Additionally, increased expression of EPHB4 has been observed in several tumors including breast, colon, head and neck, lung cancers, as well as PCa.^112^, ^113^, ^114^, ^115^, ^116^ Reduced expression of EPHB4 hinders the progression of PCa. Downregulation of EPHB4 decreases MYC expression, impairing GLUT3 function, disrupting glucose uptake, and lowering ATP levels which in turn triggers ER stress‐mediated immunogenic cell death (ICD).^117^ The activation or upregulation of AR signaling has been shown to play a key role in the progression of PCa. Numerous studies have focused on elucidating the molecular mechanisms that regulate AR signaling in PCa, to develop targeted therapeutics.^118^, ^119^, ^120^ While the primary focus of studies has been on regulating cell death by ER stress, ER stress can also influence AR signaling. ER stress elevates PERK expression, which activates eIF2α, leading to increased ATF4 levels. This, in turn, suppresses AR expression during PCa treatment.^121^ However, inducing ER stress as a treatment for PCa presents a challenge. ER stress in PCa elevates the levels of IL‐6, IL‐23p19, and TNF‐α, which can alter the tumor microenvironment. This inflammation may, in turn, promote tumorigenesis.^122^ However, the issue extends beyond inflammation and cytokine production. ER stress can also trigger the release of exosomes from PCa cells, which activate the PI3K/Akt signaling pathway, leading to increased PD‐L1 expression in macrophages.^123^ Given that PD‐L1 plays a role in immune evasion,^119^, ^124^ further investigation is needed to explore the interaction between ER stress and PD‐L1 in regulating immune responses.

Most studies have concentrated on how ER stress regulates PCa progression. However, ER stress has also been linked to the response of PCa cells to therapy. Given its role in promoting cell death, particularly apoptosis, it is evident that inducing ER stress can enhance the effectiveness of chemotherapy. For example, combining quercetin with paclitaxel increases ROS levels and triggers ER stress, leading to apoptosis and inhibiting PCa cell proliferation. This induction of ER stress improves the sensitivity of PCa cells to docetaxel chemotherapy.^125^ Thus, the role of ER stress in PCa is multifaceted, influencing various aspects of tumor cell behavior. This complex mechanism warrants further attention and investigation.

### 3.2 Pharmacological therapeutics

The pharmacological regulation of ER stress in PCa has opened new possibilities for treating the disease. This can be achieved by increasing ROS levels and other factors to induce ER stress or by modulating ER stress‐related pathways such as PERK, IRE1, and ATF6. Both phytochemicals and chemotherapy drugs have been employed to regulate ER stress in PCa treatment. While ER stress can lead to protective autophagy which may promote PCa progression, severe ER stress can trigger cell death thereby reducing PCa progression. Natural products have long served as a valuable source of anticancer drugs and therapeutic compounds, frequently being used to treat various disorders for centuries.^126^, ^127^ It is estimated that nearly 50% of currently used anticancer drugs are derived from natural sources.^128^ Plumbagin (PLB), a naturally occurring naphthoquinone isolated from *Plumbago zeylanica*, has demonstrated anticancer activity in both in vitro and in vivo studies.^129^, ^130^, ^131^ PLB has shown promise in treating PCa by regulating ER stress. It inhibits cell proliferation and promotes apoptosis. By increasing ROS production, PLB induces ER stress, which sensitizes PCa cells to undergo apoptosis.^132^ The increase in ROS levels can lead to ER stress. However, tumor cells have developed mechanisms to evade ROS‐mediated cell death, primarily by reducing ROS production. In many cancers, the upregulation of Nrf2 decreases ROS levels, promoting tumorigenesis and contributing to drug resistance. Consequently, enhancing ER stress‐mediated apoptosis can be achieved by targeting the Nrf2 pathway. In PCa, Nrf2 overexpression prevents ER stress and oxidative damage. Salinomycin downregulates Nrf2 expression, thereby increasing oxidative damage and ER stress, which accelerates apoptosis in PCa cells. This induction of ER stress through salinomycin and Nrf2 downregulation is evidenced by the overexpression of ATF4 and CHOP.^133^ The role of ROS in triggering ER stress is demonstrated by the fact that the use of N‐acetyl cysteine, a ROS scavenger, reduces ER stress and chelerythrine‐induced apoptosis in PCa cells.^134^

Chrysin, a natural compound commonly used in cancer treatment, has shown effectiveness in PCa therapy by downregulating HIF‐1α, thereby inhibiting tumor cell progression under hypoxic conditions.^135^ Chrysin has demonstrated potential in regulating ER stress for PCa treatment. It induces the loss of mitochondrial membrane potential and, by increasing ROS and lipid peroxidation, reduces tumor cell viability. Additionally, chrysin activates the UPR sensors by upregulating PERK, eIF2α, and GRP78, promoting cell death in PCa cells. Furthermore, chrysin decreases PI3K/Akt levels while upregulating MAPK and ERK1/2 pathways, thereby inhibiting PCa progression.^136^ A limitation of this study is the insufficient understanding of the role of ER stress and its associated sensors in regulating apoptosis. The effects of chrysin on ER stress and mitochondrial dysfunction have been evaluated independently, but their connection undoubtedly requires further investigation.

The relationship between ER stress and autophagy has recently gained attention. Autophagy is a cellular process responsible for eliminating toxic agents and degrading aged macromolecules. While it plays a crucial role in maintaining cell homeostasis, the activation of autophagy is also significant in cancer. The role of autophagy has been a topic of debate for years, and it is now recognized that its function is context dependent. In certain types of cancer, autophagy may have oncogenic effects. Abnormal activation of autophagy can influence tumor progression and contribute to drug resistance.^137^, ^138^ Growing evidence has shown a connection between ER stress and autophagy, highlighting the potential of ER stress to stimulate autophagy.^139^, ^140^ The role of ER stress‐mediated autophagy can be either pro‐survival or pro‐death. In PCa, the use of GZ17–6.02 enhances AMPK expression, which upregulates ULK1, leading to autophagic cell death. A combination of GZ17–6.02 and olaparib further increases protein kinase R levels, inactivating eIF2α through phosphorylation and inducing ER stress‐mediated autophagy. Inhibition of ER stress has been shown to reduce autophagosome formation and limit cancer suppression.^141^

**FIGURE 2:**
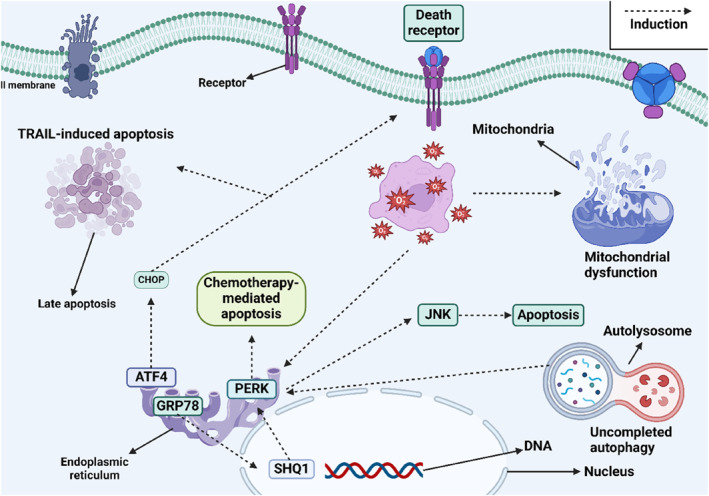
An ER stress mechanism in PCa involves elevated CHOP levels, which trigger both intrinsic and extrinsic apoptosis pathways. Increased ROS production can lead to mitochondrial dysfunction, further amplifying ER stress. UPR sensors, such as PERK, contribute to enhancing chemotherapy‐induced apoptosis. Additionally, GRP78 promotes SHQ1 transcription, resulting in PERK overexpression (Biorender.com). ER, endoplasmic reticulum.

Advances in biology have led to a greater understanding of the role epigenetic factors play in regulating biological processes. Numerous studies have demonstrated that microRNAs (miRNAs), which are short, single‐stranded, endogenous noncoding RNAs, can regulate ER stress.^142^, ^143^ Several drugs possess epigenetic functions and regulate ER stress by influencing miRNAs. For example, metformin increases the levels of miR‐708‐5p, which downregulates NNAT, leading to ER stress‐induced apoptosis and reduced viability of PCa cells.^144^ According to published reports, lncRNAs and circRNAs also play a role in regulating PCa progression.^145^, ^146^ Therefore, future studies should focus on how drugs modulate these noncoding RNAs to influence ER stress. Table 1 summarizes the role of ER stress in PCa, while Figure 2 illustrates ER stress mechanisms in PCa. The regulatory mechanisms employed by pharmacological compounds in modulating ER stress are outlined as follows: (A) the most common method involves increasing ROS levels to induce ER stress and promote apoptosis in tumor cells, (B) pharmacological compounds that induce mitochondrial dysfunction may also enhance ROS production, leading to ER stress, (C) another approach is the regulation of UPR system sensors, including IRE1, PERK, and ATF6, where their upregulation can trigger ER stress, and (D) pharmacological compounds can induce cytotoxic autophagy through ER stress but attention should also be given to ER stress‐mediated pro‐survival autophagy.

**TABLE 1:** A summary of ER stress in PCa.

Therapeutic compound	Nature of compound	Molecular profile	Highlight	References
Demethylzeylasteral (T‐96)	Natural product (isolated from tripterygium wilfordii)	‐	Demethylzeylasteral (T‐96) increases ROS levels to mediate ER stress while it impairs autophagy to finally stimulate the extrinsic pathway of apoptosis	147
Polyalthia longifolia extract	Natural product (plant extract).	‐	Polyalthia longifolia extract stimulates ER stress‐mediated apoptosis through regulation of BiP	148
Taxanes	Natural product (isolated from taxus species).	CHOP	Taxanes induce the ER stress to enhance sensitivity to TRAIL‐mediated apoptosis	149
Proscillaridin A	Natural product (isolated from urginea maritima).	‐	Increasing ROS levels and induction of ER stress	150
Isoalantolactone	Natural product (isolated from inula helenium).	JNK	Isoalantolactone enhances ROS generation to induce ER stress and JNK pathway in cisplatin sensitivity	147
‐	‐	SHQ1	GRP78 and ER sensors increase the transcriptional activation of SHQ1 to upregulate PERK for increasing chemotherapy‐mediated apoptosis	151
‐	‐	EDEM3	Downregulation of EDEM3 increases PERK, ATF6 and IRE1 levels in ER stress to reverse drug resistance	152
Norcantharidin Paclitaxel	Norcantharidin—synthetic compound (analog of cantharidin).	SIRT7	p‐eIF2α/ATF4/CHOP/cleaved‐PARP expression through SIRT7 downregulation to increase ER stress‐mediated apoptosis	153
	Paclitaxel—natural product (isolated from taxus species).			
JI017	Synthetic compound (research compound; likely synthetic).	‐	JI017 enhances ROS generation to mediate ER stress	154
CWP232291	Synthetic compound (research compound).	CHOP	Upregulation of CHOP to induce ER stress	155
Aminosteroid RM‐581	Synthetic compound (designed in the lab).	‐	ER stress‐mediated apoptosis to disrupt the cancer progression	156
Isoalantolactone	Natural product (isolated from inula helenium).	STAT3	STAT3 downregulation and ROS‐mediated ER stress in impairing PCa progression	157
δ‐Tocotrienol	Natural product (part of the vitamin E family, found in certain plant oils).	JNK	ER stress and increasing JNK levels in cancer therapy	158
Clofoctol and sorafenib	Synthetic compounds (clofoctol is a synthetic antibiotic; sorafenib is a synthetic anticancer drug).	PERK, IRE1 and ATF6	Stimulation of ER stress in reducing proliferation	159
Fenofibrate	Synthetic compound (lipid‐lowering drug).	PERK and IRE1	The uncompleted autophagy mediates ER stress	160
Aromatic monophenols from cinnamon bark	Natural products (derived from cinnamon bark).	‐	Stimulation of ER stress, downregulation of FoxM1 and induction of apoptosis	161
LCC03	Synthetic compound (research compound).	PERK	LCC03 stimulates ER stress to increase PERK levels, causing autophagic cell death	162

Abbreviation: ER, endoplasmic reticulum.

## 4 ER STRESS IN BLADDER CANCER

Bladder cancer (BCa) is one of the most prevalent types of urological cancer.^163^ BCa is classified into the following two types: nonmuscle invasive BCa (NMIBC) and muscle invasive BCa (MIBC), with NMIBC accounting for 75% of cases. The 5‐year survival rate for MIBC is 40%.^150^ The incidence of BCa is rising at an alarming rate, with 400,000 new cases diagnosed and 165,000 deaths occurring annually.^164^ The incidence of BCa is 3 to 4 times higher in males than in females making it the 7th most common malignancy in males and the 17th in females.^165^ Despite the lower incidence rate in females the prognosis for BCa is better in males. The exact reason for this disparity remains unclear.^165^

The most common subtype of BCa is transitional cell carcinoma, accounting for 90% of cases. TCC has two variants, nested and micropapillary. Other subtypes include squamous cell carcinoma and adenocarcinoma, with incidence rates of 5% and 1%, respectively. In regions with a high prevalence of schistosomiasis, such as South America, the Caribbean, East Asia, and the Middle East, SCC prevalence reaches around 75%.^166^ The prognosis of BCa is primarily determined by tumor invasiveness (T) and grade (G). According to the World Health Organization grading system, BCa is classified as well‐differentiated (G1), moderately differentiated (G2), or poorly differentiated (G3). This histological classification further divides BCa into low‐ and high‐grade diseases. High‐grade tumors are classified as either MIBC (≥T2) or NMIBC (Tis, Ta, T1) based on the extent of tumor invasion into the bladder's muscularis propria.^167^

The highest incidence of BCa is found in Lebanese females while European countries and North America report the lowest rates of the disease.^168^ Aging enhances the risk of BCa development.^169^ Conventional treatments for BCa, such as chemotherapy and surgery, have encountered significant challenges. The emergence of drug resistance has resulted in treatment failure for many patients.^170^ As a result, natural products have been proposed as alternative and complementary therapies for BCa.^171^ Additionally, nanoparticles have emerged as novel therapeutic modalities for drug and gene delivery,^172^ bioimaging,^173^, ^174^ and augmenting immunotherapy.^175^ The immune checkpoint inhibitors have been also introduced for the suppression of BCa progression.^176^ The primary challenge lies in the aggressive nature of BCa, necessitating a deeper understanding of the mechanisms driving its progression. The ability of BCa cells to tolerate stress contributes to drug resistance ^177^; however, inducing oxidative damage can inhibit BCa cell proliferation.^178^ Immune checkpoint inhibitors have also been introduced as a strategy to suppress BCa progression.

### 4.1 Progression and drug resistance

Regulatory pathways linked to ER stress can influence tumorigenesis. The interplay between ER stress and autophagy competence determines how BCa cells respond to fluid shear stress. Exposure to fluid shear stress in BCa cells triggers ER stress and decreases lysosomal movement. Additionally, ER stress can impact actin organization in BCa cells.^179^ In response to severe ER stress BCa cells typically undergo apoptosis. This process is tightly regulated and involves increased CHOP expression, Bim accumulation in the ER, and activation of caspase‐3. As a UPR sensor, IRE1 activates the TRAF2/ASK1 axis, triggering the JNK pathway to induce apoptosis in BCa cells.^180^ Excessive ROS production in BCa cells can lead to mitochondrial dysfunction by causing a loss of mitochondrial membrane potential, which in turn elevates caspase‐9 and caspase‐3 levels, accelerating cell death. Additionally, ROS stimulates ER stress by upregulating CHOP thereby inducing apoptosis in BCa. During ROS generation, there is also an increase in Nrf2 accumulation in the nucleus.^181^ Since Nrf2 plays an antioxidant role and is involved in the tumorigenesis of BCa,^182^ its downregulation and the resulting effects on ER stress warrant further investigation.

While increased ROS production is recognized as a key driver of ER stress in BCa, other factors also play a role. The primary trigger for ER stress is the accumulation of misfolded or unfolded proteins. A combination of ritonavir and RTS‐V5 can lead to the buildup of ubiquitinated proteins, inducing ER stress both in vitro and in vivo.^183^ ICD has emerged as a form of programmed cell death in which the release of DAMPs from dying cells is recognized by antigen‐presenting cells, triggering immune responses that promote cell death. Chemotherapy, radiotherapy, and other targeted antitumor therapies have the capacity to induce ICD.^184^ The induction of ICD can enhance the effectiveness of immunotherapy. CALR is a key contributor to ICD in BCa. Interestingly, downregulation of CALR triggers ER stress and decreases the survival rate of BCa cells, suggesting that distinct types of ICD exist in BCa, with some linked to poor prognosis. Thus, inhibiting the ICD‐related gene CALR can promote ER stress and reduce BCa cell viability.^185^

Tumor cells secrete extracellular vesicles (EVs) at higher levels than normal cells, and these EVs play a role in regulating tumorigenesis by transferring various cargoes that influence survival,^186^ angiogenesis,^187^ and immune responses.^188^, ^189^ Additionally, EVs have the potential to induce carcinogenesis.^190^, ^191^ Normal urothelial cells can be transformed into neoplastic cells through exposure to EVs. Inhibiting EV uptake by these normal cells disrupts the neoplastic transformation process. The transformed cells exhibit several traits, including increased genomic instability, loss of cell–cell contact inhibition, and enhanced migratory ability. Additionally, they undergo morphological changes such as expanded cytoplasm, abnormal ER structure, and smaller mitochondria. Exposure to tumor‐derived EVs activates the UPR system, and sustained UPR activation favors the survival branch. These cells show upregulation of IRE1, NF‐κB, and leptin, while losing CHOP, a proapoptotic protein. Inhibiting ER stress hinders the ability of EVs to mediate the neoplastic transformation of normal urothelial cells.^192^ This study demonstrated that while severe ER stress can lead to cell death in BCa, chronic and prolonged ER stress is a key factor in tumorigenesis, driving the transformation of normal cells into neoplastic ones. The role of ER stress in regulating drug resistance in BCa has also been investigated. Several studies have revealed that, although gemcitabine is commonly used in BCa chemotherapy, alternative molecular interactions—such as Akt upregulation and dysregulation of the LINC00839/SOX5 axis—can contribute to resistance.^193^, ^194^ P4HB upregulation is associated with poor prognosis in BCa and contributes to gemcitabine resistance. Conversely, P4HB downregulation activates the PERK/eIF2α/ATF4/CHOP axis, related to ER stress, promoting apoptosis and enhancing gemcitabine sensitivity in BCa.^195^ Although studies have explored the mechanisms of ER stress in BCa, many aspects remain to be clarified. For example, EVs are thought to induce sustained ER stress during the neoplastic transformation of normal cells, but the molecular mechanisms behind this hyperactivation require further investigation. Additionally, while stemness contributes to tumorigenesis and drug resistance, the link between ER stress and stemness needs more examination. Furthermore, the relationship between ER stress and epithelial‐mesenchymal transition (EMT) in BCa should also be explored in greater detail.

### 4.2 Pharmacological therapeutics

In BCa research, the primary focus has been on using pharmacological compounds to induce ER stress. Pharmacological treatments have shown significant promise and are actively used in clinical settings to treat patients. Combination therapy has emerged as a highly effective approach in reducing BCa progression. For example, a combination of nelfinavir and ritonavir induces ER stress by upregulating GRP78, ER‐resident protein 44, and endoplasmic oxidoreductase‐1‐like protein, leading to the elimination of BCa cells.^196^

β‐asarone (β‐as), a compound isolated from *Acorus calamus* has been studied for its distribution and absorption properties.^197^ *β*‐asarone can penetrate the blood‐brain barrier, making it a potential treatment for Alzheimer's disease.^198^ Additionally, *β*‐asarone has been extensively used in the treatment of various human cancers.^199^, ^200^, ^201^, ^202^ The potential of *β*‐asarone in BCa treatment is linked to its ability to regulate ER stress, demonstrating that ER stress modulation can influence the invasion and metastasis of BCa cells. *β*‐asarone inhibits EMT and metastasis in PCa cells while promoting ATF6 expression, enhancing Golgi cleavage and nuclear localization. By inducing ER stress, *β*‐asarone impairs the metastasis of BCa cells by suppressing EMT.^203^ Since EMT plays a role in promoting drug resistance,^204^, ^205^ further studies should more thoroughly investigate how ER stress regulates EMT and its connection to the therapy resistance of BCa cells.

In earlier sections, it was noted that chrysin induces ER stress to target PCa. Its role in BCa treatment has also been explored. Chrysin increases the levels of caspase‐3 and ‐9 to trigger apoptosis while reducing Bcl‐2, Mcl‐1, and Bcl‐xl levels to accelerate cell death. Additionally, chrysin upregulates PERK and ATF4, promoting ER stress. By enhancing ROS generation, chrysin further induces ER stress, which can hinder BCa progression.^206^

A key limitation of current studies examining the role of ER stress in BCa therapy and other urological cancers is the insufficient focus on cell death mechanisms. Most research has concentrated on the regulation of ER by pharmacological compounds, while overlooking the role of mitochondria in promoting cell death. For instance, flaccidoxide‐13‐acetate has been shown to mediate several changes in BCa cells during cell death induction, including the following: (A) upregulation of JNK to promote apoptosis, (B) overexpression of Bad and Bax, along with downregulation of Bcl‐Xl and Bcl‐2, triggering caspase‐3‐driven apoptosis, and (C) ER stress induction, which elevates GRP78/PERK levels to activate the ATF4/CHOP axis, leading to apoptosis.^207^ ER stress can impact both intrinsic and extrinsic apoptosis pathways. Therefore, further research is needed to assess how ER stress‐induced apoptosis interacts with mitochondrial function. With the extrinsic pathway ER stress has been linked to increased sensitivity to TRAIL‐mediated cell death.^208^ The interaction between ER stress and mitochondria in BCa cells requires further investigation. Notably, studies have shown that thymoquinone induces ER stress through CHOP and GRP78 in BCa treatment. Decreased CHOP expression leads to an increase in Bcl‐2 levels, preventing apoptosis, demonstrating that ER stress‐induced mitochondrial dysfunction plays a role in triggering apoptosis.^209^ However, additional underlying mechanisms remain to be identified. In the activation of apoptosis via the mitochondrial or intrinsic pathway, the upregulation of caspase‐12 is considered crucial. Licochalcone increases the expression of GRP78 and CHOP to induce ER stress, which subsequently leads to caspase‐12 upregulation, triggering apoptosis through the mitochondrial pathway in BCa cells.^210^

Elevated ROS production and altered redox balance can drive tumorigenesis.^211^ Excessive ROS production triggers biochemical and molecular changes that can influence cancer initiation, promotion, progression, and contribute to drug resistance.^211^, ^212^ A key finding in numerous studies is that increased ROS levels enhance ER stress, leading to apoptosis. In a recent experiment, Jolkinolide B was found to promote ROS production, inducing ER stress and activating the ERK pathway, ultimately triggering paraptosis in BCa cells. This elevated ROS production is linked to a reduction in TrxR1 levels and the inhibition of GSH.^213^

**FIGURE 3:**
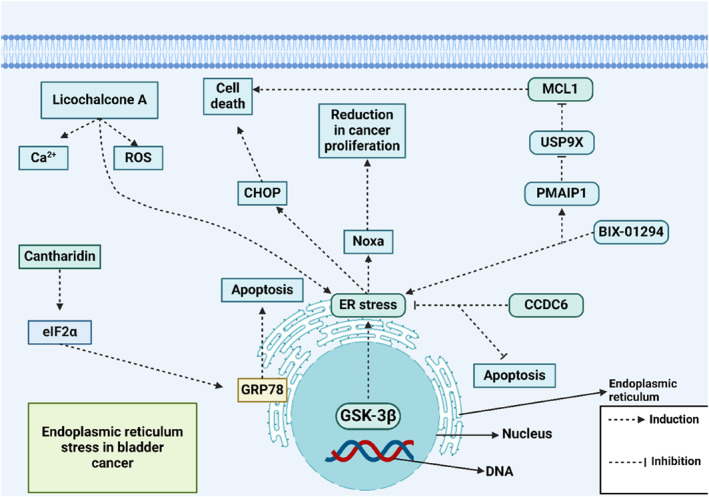
The ER stress in BCa. Licochalcone A increases Ca2+ and ROS levels, triggering ER stress. Additionally, GSK‐3β enhances ER stress, upregulating Noxa levels to reduce proliferation and induce apoptosis. Cantharidin raises eIF2α levels, promoting GRP78 expression, which mediates apoptosis, while CHOP upregulation further stimulates apoptotic processes (Biorender.com). ER, endoplasmic reticulum.

In conclusion, the pharmacological regulation of ER stress offers promising new insights for the treatment of BCa. Figure 3 and Table 2 summarize ER stress in BCa, with key findings as follows: (A) Increased ROS generation induces ER stress, leading to the elimination of BCa cells. (B) ER stress triggers both apoptosis and paraptosis in BCa cells. (C) The role of ER stress‐mediated autophagy in BCa cells requires further investigation to determine whether it acts as a carcinogenic or anti‐carcinogenic factor. (D) ER stress‐related sensors control both intrinsic and extrinsic apoptosis pathways and regulate mitochondrial functions. (E) Pharmacological compounds can disrupt redox balance in tumor cells by downregulating GSH and other factors, such as TrxR1, to enhance oxidative damage‐induced ER stress.

**TABLE 2:** The role of ER stress in BCa.

Pharmacological compound	Nature of compound	Molecular profile	Highlight	References
Cantharidin	Natural product (derived from blister beetles, specifically mylabris species).	eIF2α and Grp78	Cantharidin increases levels of cantharidin to mediate ER stress, causing apoptosis	214
Melatonin valporic acid	Melatonin ‐ natural product (a hormone produced in the pineal gland of animals, though it can also be synthesized in laboratories).	ATF6, IRE1, EDEM1 and ERdj4	Upregulation of ER stress‐related factors including ATF6, IRE1, EDEM1, and ERdj4	215
	Valproic acid ‐ synthetic compound (a pharmaceutical drug used as a mood stabilizer and anticonvulsant).		Controlling apoptosis, necrosis, and autophagy	
Stevioside	Natural product (a sweetener derived from the leaves of the plant stevia rebaudiana).	GSK‐3β	Stevioside stimulates ER stress and GSK‐3β to increase noxa levels, suppressing cancer proliferation	216
‐	‐	CCDC6	The downregulation of CCDC6 stimulates ER stress‐mediated apoptosis and promotes response of BCa cells into oncolytic virus M1	217
‐	‐	‐	The transfer of IFN‐α stimulates ER stress‐mediated anticancer function	218
Licochalcone A	Natural product (isolated from the roots of glycyrrhiza inflata).	ROS	Licochalcone a promotes Ca^2+^ and ROS levels to mediate ER stress‐induced apoptosis	219
		Ca^2+^		
‐	‐	Noxa	Proapoptotic function of ER stress is facilitated through upregulation of noxa	220
BIX‐01294	Synthetic compound (a selective inhibitor of G9a histone methyltransferase, developed in the lab).	PMAIP1/USP9X/MCL1	BIX‐01294 stimulates ER stress and promotes levels of PMAIP1 through DDIT3 overexpression	221
			Reducing USP9X levels by BIX‐01294 downregulates MCL1 expression to induce apoptosis	
Ethanol extract of pomegranate fruit	Natural product (extracted from the fruit of Punica granatum).	CHOP	Ethanol extract of pomegranate fruit increases CHOP and Bip levels to mediate apoptosis	222
		Bip		

Abbreviation: ER, endoplasmic reticulum.

## 5 ER STRESS IN RENAL CANCER

Renal cell carcinoma (RCC) is one of the most common cancers worldwide, with rising prevalence, accounting for 400,000 new cases and 180,000 deaths each year.^223^, ^224^ Approximately 30% of RCC cases are diagnosed at advanced or metastatic stages, where the 5‐year survival rate is only 10%.^225^ The introduction of targeted therapies and immune checkpoint inhibitors has enhanced the effectiveness of RCC treatment. However, a deeper understanding of RCC etiology is necessary to further improve treatment outcomes, prognosis, and survival rates.^226^ In the United States, RCC represents 5% of all cancer diagnoses in males and 4% in females.^227^ Because the majority of RCC cases are diagnosed at advanced stages treating the disease presents several challenges.^228^ Following surgical resection up to 40% of patients experience RCC recurrence. Additionally, RCC patients often develop resistance to standard treatments like chemotherapy and radiotherapy. The response of RCC to immunotherapeutic agents such as INF‐α and IL‐12 is generally poor. The identification of molecular and cytogenetic biomarkers has led to improved classification of RCC.^229^ As a result, new therapeutic approaches, including RTK and mTOR kinase inhibitors, can be employed for the treatment of metastatic RCC.^229^, ^230^ However, the response of RCC to therapy remains unpredictable and its prognosis is generally poor.^230^ The incidence of RCC varies geographically with Europe and Australia having the highest rates. Additionally, the highest mortality rates are observed in Uruguay, Argentina, Chile, and the USA.^231^ RCC has over 10 subtypes based on histopathological and molecular profiles. The most common are clear cell renal carcinoma, accounting for 75% of cases, followed by papillary RCC (types I and II) and chromophobe RCC. The rarer subtypes make up only 1% of all cases.^232^ In recent years, the molecular interactions in RCC have gained significant importance, as understanding these changes can offer new insights for treating the disease. S1P promotes renal cancer proliferation and metastasis by stimulating EMT, with these oncogenic effects being mediated by the upregulation of S1PR3.^233^ The reduction of TGF‐β levels by c‐Ski can contribute to the progression of RCC.^234^ Additionally, ELOVL2 inhibits apoptosis, promoting the survival of renal cancer cells.^235^ The dysregulation of epigenetic factors, such as lncRNA CYTOR and RCAT1, can influence the proliferation and metastasis of RCC cells.^236^, ^237^ The molecular interactions described are crucial in RCC tumorigenesis. The following sections will explore the role of ER stress in RCC progression, highlighting its importance for further research.

### 5.1 Progression and drug resistance

ER stress plays a role in the development of chemoresistance in RCC. Sunitinib, a common treatment for RCC, triggers ER stress marked by increased PERK expression. PERK overexpression during ER stress elevates levels of proinflammatory factors such as IL‐6, IL‐8, and TNF‐α, which have oncogenic functions, and activates the TRAF2‐mediated NF‐κB pathway as a pro‐survival mechanism to reduce cell death. Consequently, inhibiting PERK increases the sensitivity of RCC cells to sunitinib chemotherapy.^238^ Reduced Par‐4 expression can induce ER stress, which enhances TRAIL‐mediated apoptosis in RCC cells.^239^ However, most studies have focused on the therapeutic regulation of ER stress in RCC, as discussed below.

### 5.2 Pharmacological therapeutics

The induction of apoptosis is a common strategy for reducing tumorigenesis in renal cancer. Nelfinavir promotes apoptosis and decreases colony formation by triggering ER stress and elevating DR4 and DR5 levels, leading to TRAIL‐induced apoptosis.^240^ To enhance the anticancer efficacy of Nelfinavir, its combination with panobinostat has been suggested. Together, Nelfinavir and panobinostat stimulate apoptosis and inhibit cell proliferation through a synergistic mechanism involving ER stress and histone acetylation. Additionally, Nelfinavir increases AMPK expression, leading to mTOR downregulation. The induction of ER stress is crucial for this anticancer effect, as the use of cycloheximide, a protein synthesis inhibitor, reduces apoptosis.^241^ The induction of ER stress following combination therapy is evidenced by the upregulation of GRP78 and HSP70.^242^ In certain cases, the combination therapy induces both ER stress and histone acetylation by downregulating HDAC, thereby inhibiting the progression of renal cancer.^243^ The upregulation of HDAC6 promotes the accumulation of ubiquitinated proteins and ER stress, which together work synergistically to hinder cancer progression.^244^ Furthermore, the combination therapy can induce ER stress and downregulate mTOR in the treatment of renal cancer.^245^ The reports provide detailed insights into how these therapies stimulate ER stress in renal cancer.^246^, ^247^

**FIGURE 4:**
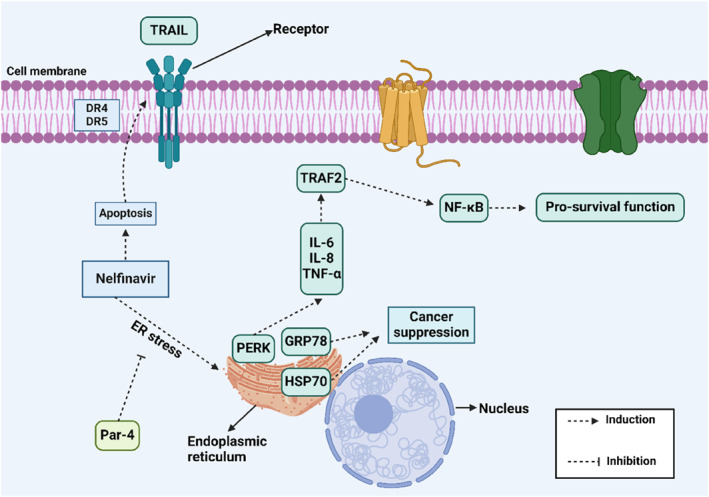
The role of ER stress in renal cancer involves several key mechanisms. ER stress activation increases the levels of DR4 and DR5, triggering the extrinsic apoptotic pathway. Additionally, the downregulation of Par‐4 stimulates ER stress. ER stress and PERK upregulation can elevate IL‐6, IL‐8, and TNF‐α levels, leading to increased TRAF2 expression. This, in turn, upregulates NF‐κB, promoting cancer progression (Biorender.com). ER, endoplasmic reticulum.

Figure 4 illustrates the role of ER stress in RCC, while Table 3 summarizes the prognostic significance of key ER stress markers in urological cancer patients. However, there are several limitations to consider.

A) The underlying mechanisms that regulate ER stress have been largely overlooked. Current discussions focus primarily on the role of ER stress in enhancing drug sensitivity and mediating TRAIL‐induced apoptosis. However, ER stress also plays a role in regulating cancer proliferation, metastasis, and stemness and these underlying mechanisms need further exploration.
B) While the function of ER stress in regulating proinflammatory cytokines has been mentioned, its effect on the tumor microenvironment, particularly the polarization of macrophages and immune cell infiltration, requires further investigation.
C) ER stress has significant potential to induce autophagy. The role of ER stress‐mediated autophagy in RCC should be thoroughly evaluated.
D) The interaction between ER stress markers such as CHOP and mitochondrial dysfunction in RCC needs to be further emphasized.
E) Various drugs and phytochemicals have been used to induce ER stress, but the molecular mechanisms behind their actions have not been adequately addressed.
F) Since molecular regulators of ER stress have been identified, the development of small molecules targeting these regulators could be a promising avenue for future research.

**TABLE 3:** The role of key ER stress markers in the prognosis of urological cancers.

ER stress marker or related genes	Cancer	Association with prognosis	Reference
ERLIN2 and CDK5RAP3	PCa	ER stress‐related factors affecting prognosis and immune infiltration	
		ERLIN2 as an anti‐oncogene and CDK5RAP3 as a pro‐oncogene	
ATF6α, PERK, and IRE1α	PCa	Stimulation of UPR mediates poor prognosis	248
ATP2A3, STIM2, VWF, and P4HB	BCa	The higher ER stress scores, based on these genes, correlate with worse prognosis and are linked to significant immune cell infiltration and higher immune scores.	249
HSP90B1	BCa	High expression of HSP90B1 is associated with poor prognosis and is closely related to PD1, indicating its potential as a prognostic biomarker and therapeutic target for cancer immunotherapy.	250
AL355488.1, AL035461.2, MAFG‐DT, AC008735.2, MIR200CHG, and KRT7‐AS	BCa	These lncRNAs were found to be significantly associated with prognosis, forming the basis of a risk score model that predicts overall survival in patients. Higher risk scores correlated with poorer outcomes, underscoring their potential utility in prognostic assessments and therapeutic targeting in BCa.	251
GRP78	Renal cancer	Upregulation of GRP78 and enhanced levels in serum correlate with tumor stage	252
PERK and ATF6	Renal cancer	Increase in the levels of PERK and ATF6 mediate better response to sunitinib	253

Abbreviation: ER, endoplasmic reticulum.

## 6 CONCLUSION AND PERSPECTIVES

The treatment of urological cancers has encountered significant challenges due to the development of drug resistance. Chemotherapy remains the gold standard for treating these cancers, primarily by inducing apoptosis and reducing tumor cell viability. However, the activation of alternative mechanisms has led to drug resistance by decreasing cell death and enhancing DNA damage repair. Regulating ER stress offers a promising approach for improving treatment outcomes and increasing chemosensitivity in urological cancers. By stimulating ER stress, immunotherapy can be enhanced and apoptosis can be triggered. ER stress activates both intrinsic and extrinsic apoptotic pathways, making it a potential therapeutic target.

Pharmacological induction of ER stress can enhance the suppression of urological cancers. ER stress occurs when the ER's protein folding capacity is overwhelmed by stressors such as hypoxia, nutrient deprivation, and increased protein synthesis. In PCa, BCa, and RCC, ER stress activates the UPR, which initially seeks to restore ER homeostasis but can trigger cell death if stress persists. This dual nature influences cancer cell survival, proliferation, and apoptosis.

A common mechanism across these cancers is the role of GRP78 (BiP), a key ER chaperone that supports cell survival under stress. Elevated GRP78 levels have been observed in PCa, BCa, and RCC, contributing to disease progression and chemotherapy resistance. For example, in PCa, GRP78 enhances AR activity and promotes survival pathways, while in BCa and RCC, it aids cellular adaptation to hypoxic conditions, which are typical in solid tumors.

Another shared feature is the activation of the PERK pathway, a UPR branch that leads to eIF2α phosphorylation, reducing general protein synthesis while selectively increasing ATF4 translation. This can either promote survival or induce apoptosis. In all three cancer types, this pathway has been linked to therapy resistance and cancer cell survival under harsh conditions.

However, the specific effects of ER stress vary among PCa, BCa, and RCC due to differences in their microenvironments, genetic profiles, and therapy sensitivities. In PCa, ER stress is closely tied to AR signaling, which is crucial for disease development and progression. UPR activation can modulate AR signaling, boosting the expression of genes that drive cancer growth. Additionally, ER stress‐associated autophagy protects PCa cells, allowing them to survive hormonal therapy.

In BCa, ER stress often results in higher NLRP3 inflammasome expression, promoting inflammation and potentially encouraging tumor growth and immune evasion. BCa cells may exploit ER stress responses to resist the cytotoxic effects of urinary toxins and chemotherapy.

In RCC, hypoxia is particularly relevant, as RCC tumors are frequently highly hypoxic, intensifying ER stress and activating unique adaptive mechanisms. HIFs, stabilized under low oxygen conditions, interact with UPR pathways, especially the IRE1‐XBP1 axis, promoting cell survival and angiogenesis. This hypoxia‐driven ER stress response is more pronounced in RCC than in other urological cancers.

The prognostic value of ER stress markers also varies. High GRP78 expression is associated with poor prognosis in PCa and RCC but has a more variable impact in BCa. Therapeutic targeting of ER stress pathways differs as well; for instance, PERK inhibitors may be effective in RCC due to its reliance on hypoxia‐driven ER stress but may be less effective in PCa, where AR signaling dominates. Despite significant advancements in the field, several limitations and opportunities for future research remain:

A) Many ER stress‐related mechanisms have been overlooked in urological cancers. For instance, while the role of ER stress in regulating inflammation has been explored in RCC, it has been largely ignored in PCa and BCa. Additionally, ER stress has a strong potential for inducing autophagy, which has not been thoroughly investigated in urological cancers.
B) The interaction between ER stress and mitochondrial dysfunction has been established, but further research is needed on how cytochrome C release during mitochondrial dysfunction might mediate necrosis in urological cancers.
C) PERK upregulation during ER stress has been shown to modulate angiogenesis through VEGF overexpression. The role of ER stress in regulating angiogenesis in urological cancers, however, remains underexplored and warrants further investigation.
D) ER stress has demonstrated a modulatory effect on macrophage polarization in other diseases, a topic that needs to be studied in the context of urological cancers.
E) The status of ER stress during hypoxia in urological cancers should be a focus of future research, as this relationship is not well understood.
F) Regarding the application of pharmacological compounds, some therapeutics in clinical trials have faced challenges due to poor pharmacokinetic profiles. Therefore, utilizing nanoparticles for targeted therapeutic delivery and the regulation of ER stress is a promising avenue for future studies.

## AUTHOR CONTRIBUTIONS

Najma Farahani, Mina Alimohammadi, Mehdi Raei: Writing—original draft preparation. Kiavash Hushmandi: Conceptualization. Salman Daneshi, Alireza Razzaghi: Investigation. Noushin Nabavi, Amir Reza Aref: Writing—review and editing. Mehrdad Hashemi, Afshin Taheriazam, Kiavash Hushmandi: Supervision.

## CONFLICT OF INTEREST STATEMENT

The authors declare no conflicts of interest.

## ETHICS STATEMENT

Not applicable.

## CONSENT FOR PUBLICATION

Not applicable.
